# Osteonecrosis in sickle cell disease: Contemporary orthopaedic practice and outcomes across African healthcare settings

**DOI:** 10.1051/sicotj/2026008

**Published:** 2026-04-08

**Authors:** Emmanuel Olusola Oladeji, Oluwatobi Olayode, Abdulahi Zubair, Vernon Ipomai, Oluwafemi Olayinka, Patrick Okonkwo, Onyeka Omerenma, Gregory Okhifun, Oghofori Obakponovwe

**Affiliations:** 1 Trauma and Orthopaedics Department, Surgery Interest Group of Africa Km 43, Lekki-Epe Expressway Ibeju-Lekki Lagos State 105101 Nigeria; 2 University of Nairobi, Faculty of Health Sciences Nairobi Kenya

**Keywords:** Osteonecrosis, Avascular necrosis, Sickle cell disease, Africa, Low-resource settings, Global surgery

## Abstract

*Background*: Osteonecrosis is a disabling complication of sickle cell disease (SCD), with a disproportionate burden in Africa, where healthcare resources are limited. Despite this, the region remains underrepresented in the literature on SCD-related osteonecrosis. This scoping review synthesises current evidence on the epidemiology, management practices, and outcomes of SCD-related osteonecrosis in African healthcare settings. *Methods*: Following PRISMA-ScR guidelines, we systematically searched MEDLINE, Embase, Web of Science, Google Scholar, and African Journals Online through February 2025. Eligible studies reported clinical features, management, or outcomes of osteonecrosis in SCD patients in Africa. Data on demographics, staging, imaging, treatment modalities, and outcomes were narratively synthesised. *Results*: Thirty-two studies involving 779 patients met the inclusion criteria. Most were small, descriptive case series. Patients typically presented late: 85% at Ficat stage III–IV, with delays exceeding 20 years in some cases. The femoral head was affected in 98% of cases. Diagnosis relied almost exclusively on radiographs, with MRI reported in only 6% of studies. Conservative management, mainly traction and immobilisation, showed benefit in paediatric early-stage cases but was largely ineffective in adults. Joint-preserving surgeries were rarely reported but included core decompression and vascularised grafting with variable success. Arthroplasty predominated, yielding functional improvement but was technically demanding and prone to complications, particularly in SS genotype patients. *Conclusion*: Late presentation, diagnostic limitations, and reliance on salvage arthroplasty mark SCD-related osteonecrosis in Africa. Strengthening early detection, expanding capacity for joint-preserving interventions, and generating robust regionally relevant evidence are critical to improving outcomes in this high-burden, resource-constrained setting.

## Introduction

Sickle cell disease (SCD) comprises a group of inherited haemoglobinopathies resulting from a single β-globin mutation that produces haemoglobin S [1]. Sickled erythrocytes obstruct the microvasculature, causing recurrent vaso-occlusion, ischaemia, and progressive end-organ damage. Among its chronic complications, osteonecrosis or avascular necrosis (AVN) is particularly debilitating, arising from disrupted bone perfusion and leading to collapse, pain, and joint dysfunction [2, 3].

Historically, most children with SCD in Africa died before diagnosis, but survival has improved with newborn screening, disease awareness, and basic supportive care [4]. Consequently, more individuals now live into adolescence and adulthood, where AVN becomes increasingly prevalent, affecting up to 50% of patients, particularly the femoral and humeral heads [5–7].

SCD is globally distributed through migration, but prevalence remains highest in sub-Saharan Africa, Asia, and the Caribbean [1, 8]. High-income countries have made substantial advances in understanding and managing SCD-related AVN [9–12]. In contrast, African patients – who bear most of the global burden – face constrained health systems, scarce specialist services, high out-of-pocket costs, and pervasive inequities [13]. These systemic barriers contribute to profound disparities in outcomes compared with high-resource settings.

The rising prevalence of SCD in Africa, driven by demographic growth and the high frequency of sickle trait, predicts a parallel increase in AVN [4, 8]. Yet, despite accounting for more than 80% of global SCD births, the continent remains underrepresented in research on AVN epidemiology, management, and outcomes. This gap limits the availability of locally relevant evidence to inform clinical practice and health policy.

To address this gap, we conducted a scoping review to synthesise current knowledge on the burden, management practices, and outcomes of AVN in African patients with SCD. By consolidating available evidence, we aim to inform clinicians, policymakers, and researchers, strengthen care pathways, and highlight priorities for future research.

## Methods

This scoping review followed the methodological framework of Peters et al. and was reported in accordance with the PRISMA-ScR guidelines [14]. We searched MEDLINE, Embase, Web of Science, Google Scholar, and African Journals Online from inception to February 2025, using terms including *sickle cell disease*, *avascular necrosis*, *Africa,* and their variants. Reference lists of eligible articles were also screened.

Inclusion criteria comprised peer-reviewed studies reporting on the clinical features, management, or outcomes of SCD-related AVN in African healthcare settings. No restrictions were applied for age, sex, or year of publication. Studies addressing AVN of mixed aetiologies were included if they presented data specific to SCD. Abstract-only publications, reviews, and meta-analyses were excluded.

Search results were imported into the Rayyan software, with duplicates removed. Two reviewers independently screened titles, abstracts, and full texts, resolving disagreements by consensus. Data were extracted using a piloted Microsoft Excel form. Variables included publication year, study design, country, patient demographics, haemoglobin genotype, anatomical sites involved, staging, and imaging modalities. Treatment strategies, delays in care, outcomes, complications, and barriers to management were also recorded. Results were narratively synthesised to map the burden, practice patterns, and reported outcomes and barriers of SCD-related AVN across African settings.

## Results

### Study and patient characteristics

**Figure 1:**
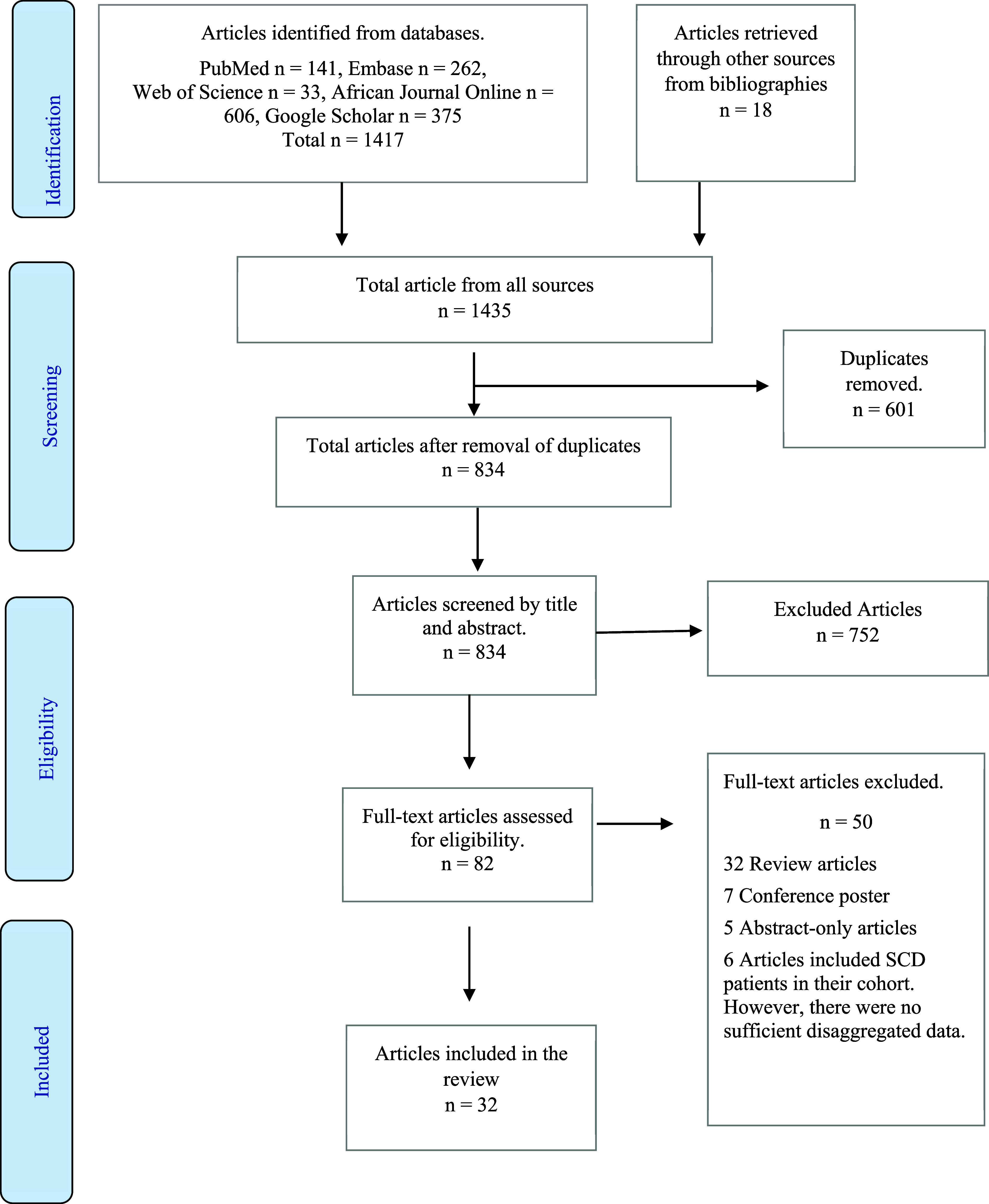
PRISMA flow chart illustrating the article selection process. SCD: Sickle Cell Disease.

Figure 1 illustrates the article selection process for this review. A total of 1435 articles initially identified were systematically screened against the predetermined inclusion criteria. Thirty-two studies, encompassing 779 patients with SCD-related AVN, met these criteria.

Half of the studies were case series, with prospective cohorts at 18%, cross-sectional designs at 12.5%, case reports at 12.5%, and case-control studies at 6% (Table 1). Sample sizes varied widely, ranging from individual case reports to series comprising up to 75 patients, reflecting the heterogeneity of the available evidence. Geographically, Nigeria contributed more than half of the included studies (17 out of 32), followed by Togo (3), Tunisia (2), and single studies from Ghana, Sierra Leone, Congo, Gabon, Morocco, Côte d’Ivoire, Senegal, Uganda, Cameroon, and Niger. This distribution underscores both the dominance of Nigerian orthopaedic research output and the limited published data from other high-prevalence regions in sub-Saharan Africa.

**Table 1:** Overview of study characteristics.

Author	Year	Study design	No. of AVN cases	Disease stage (Ficat)	Bone(s)/joint(s) affected	Management approach
Iwegbu and Fleming [15]	1985	Case Series	29	I; II; 8; III; 16; IV; 5	Femoral head	NA
Ben Dridi et al. [16]	1987	Case Series	13	NA	Femoral head, humeral head	NA
Ugbeye et al. [17]	2020	Case Series	56	I; II; III; IV; 68	Femoral head	Arthroplasty
Katchy et al. [18]	2018	Case series	21	NA	Femoral head	Arthroplasty
Ekere et al. [19]	2008	Case series	2	I; II; III; 1; IV; 1	Femoral head	Arthroplasty
Ebong et al. [20]	1985	Case series	75	NA	Femoral head	Physical immobilization
						Femoral osteotomy
Ugbeye et al. [21]	2024	Case report	1	I; II; III; IV; 2	Femoral head	Arthroplasty
Agbeko et al. [22]	2024	Cross-sectional	54	I; II; 23; III; 8; IV; 23	Femoral head	NA
Onyemaechi et al. [23]	2011	Case series	12	I; II; 2; III; 7; IV; 3	Femoral head	Joint offloading Femoral osteotomy
						Arthroplasty
Lumbardo et al. [24]	2020	Case series	3	I; II; III; IV; 3	Femoral head	Arthroplasty
Manfroni et al. [25]	2020	Case report	1	NA	Femoral head	Arthroplasty
Akinyoola et al. [26]	2007	Case series	66	II-12; III-14; IV; 40	Femoral head	NA
Knox-Macaulay [27]	1983	Prospective cohort	9	NA	Femoral head	NA
Bokolombe et al. [28]	2013	Case report	1	I; II; 1; III; IV	Femoral head	Arthroplasty
Alonge et al. [29]	2004	Case series	5	I; II; III; IV; 4	Femoral head	Arthroplasty
						
Balogun et al. [7]	2010	Prospective cohort	28	I; II – 1; III – 7; IV – 20	Femoral head	NA
						
Akakpo-Numado et al. [30]	2008	Case Series	14	I; II; III – 8; IV – 6	Femoral head	Femoral osteotomy
Mouba et al. [31]	2011	Case Series	22	I; II; III – 22; IV	Femoral head	Traction device
						Cast immobilization
						Offloading orthoses
						Physical therapy
Bennis et al. [32]	2020	Case report	1	NA	Femur shaft	Joint offloading
N'Dri et al. [33]	2000	Cross-sectional	34	III – 34	Femoral head	NA
Akinyoola et al. [34]	2008	Case control	25	I – 1; II – 7; III – 4; IV – 13	Femoral head	NA
Ouerderni et al. [35]	2023	Case Control	41	I – 7; II – 12; III – 22; IV	Femoral head	NA
Wu et al. [36]	2005	Case series	14	NA	Femoral head	Iliac bone grafting
Ebong [37]	1976	Case series	22	NA	Femoral head	Hip spica immobilization
Sene et al. [38]	2009	Case Series	38	I; II; III – 10; IV – 28	Femoral head	Arthroplasty
Ndugwa [39]	1992	Case Series	47	I; II; III; IV – 47	Femoral head	Traction device
						Cast immobilization
Ofakunrin et al. [40]	2021	Cross-sectional study	38	I; II – 8; III – 9; IV – 16	Femoral head	NA
Bahebeck et al. [41]	2004	Prospective cohort	35	I – 6; II – 4; III – 11; IV – 4	Femoral head, lumbar spine, humeral head, talar body	Core decompression
						Arthroplasty
						Arthrodesis
Mosaku et al. [42]	2015	Cross-sectional study	13	NA	NA	NA
Alabi et al. [43]	2021	Prospective cohort	27	I; II; III; IV – 38	Femoral head	Arthroplasty
						Adductor tenotomy
Bamgbade et al. [44]	2021	Prospective cohort	14	Steinberg stage VI – 14^a^	Femoral head	Arthroplasty
Lawal et al. [45]	2017	Prospective cohort	18	I; II; III – 18; IV	Femoral head	Arthroplasty

^a^The Steinberg classification was adopted in this study. NA – Not available or Not applicable.

**Figure 2:**
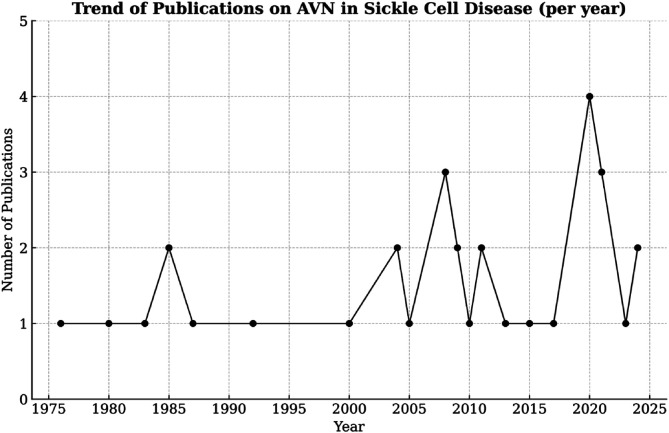
Publications on SCD-related avascular necrosis from African centres (1976 – 2025).

The studies spanned from 1976 to 2024 (Figure 2), with 57% focusing on adults and 43% including children and adolescents. Patient ages ranged from 2 to 57 years, with a slight female predominance (52%). The haemoglobin SS genotype was most common (78%), followed by SC (19%). Only a small proportion had the S/β-thal (3%) genotype. These distributions align with the known burden of severe musculoskeletal complications in homozygous patients.

### Pattern of presentation and diagnostic practices

The femoral head was affected in 98% of cases, with bilateral disease reported in 24%. Bilateral involvement was more common among SS genotype patients, although only two-thirds experienced symptoms in both hips. The most common presenting complaints included hip pain, limp, and progressive limitations in weight-bearing and mobility. Deformity and severe abduction restriction, sometimes accompanied by significant functional and sexual impairment, were frequently observed in advanced stages. Other affected anatomical sites included the humeral head [16, 31, 40, 41], the femoral diaphysis [32], the talus [41], and the lumbar spine [41], although these were rarely reported.

Symptom duration ranged from weeks to decades, with extremes including first-time presentations occurring more than 20 years after symptom onset. Most patients presented late: 52% were stage IV, 33% stage III, 12% stage II, and only 3% stage I. One study using the Steinberg classification reported that all patients were at stage VI [44]. Published before these classification systems became widespread, two studies categorised radiological diagnoses into three groups: Perthes-like lesions, osteochondritis dissecans-type lesions, or severe hip deformity [20, 37].

Plain radiographs were the most used imaging method, featuring in 97% of studies. MRI was documented in only 6%, CT in 12.5%, and bone scintigraphy in 3%. Advanced imaging was often only used when plain radiographs proved inconclusive, but it was not always accessible in these low-resource settings.

### Management approaches

#### Conservative management

Conservative management was documented in 169 patients, primarily children and adolescents [20, 23, 30–32, 37, 39, 41]. Strategies included traction (skin or Russell traction), cast immobilisation, Thomas splints, shoe lifts, orthoses, and strict activity modification (Table 2).

**Table 2:** Overview of management strategies.

Management	Number of studies reporting	Total number of patients
Conservative management^a^	8	169
Continuous traction		
Russell traction followed by hip spica	1	16
Russell traction followed by Thomas splint	1	10
Skin traction	1	9
Unspecified traction device followed by offloading orthosis	1	22
Immobilizing cast		
Alternating hip spica and knee cylinder		
Offloading with crutches or frame	2	95
	3	17
Joint-preserving surgeries^a^	6	44
Femoral/pelvic osteotomy	3	13
Core decompression	2	16
Core decompression + cementoplasty	1	3
Iliac bone grafting	1	12
Arthroplasty	14	207
Total hip arthroplasty		
Cemented	2	40
Uncemented	10	147
Hemiarthoplasty		
Bipolar	1	18
Excision arthroplasty	1	2
Arthrodesis	1	1

^a^More than one treatment modality was reported.

Ndugwa reported 16 patients with Ficat stage II disease managed with 6–12 weeks of Russell traction followed by spica immobilisation, advising transition to surgery if no improvement was seen at six weeks [39]. Akakpo-Numado et al. treated eight stage III and two stage IV patients with 30 days of traction, followed by a subsequent Thomas splint, which was used for an average of 14 months [30]. Onyemaechi et al. used skin traction in nine patients, although details were limited [23], while Mouba et al. described the use of an unspecified traction device followed by immobilisation with spica casts, offloading orthoses, or shoe lifts [31].

Other immobilisation strategies included alternating hip spica and non-weight-bearing knee cylinders, as described in two series involving 95 patients [20, 37]. Immobilisation was alternated every six weeks, with follow-up radiographs every three to six months for up to two years.

Adjuncts to conservative care emphasised pain control and lifestyle modification. Analgesia followed a stepped approach from non-opioids to NSAIDs and short-term opioids, alongside activity modification and structured rest. These measures aimed to reduce pain, preserve function, and delay surgery. In contrast, Ben Dridi et al. noted accelerated AVN progression in patients misdiagnosed with rheumatoid arthritis who received intra-articular corticosteroids [16].

#### Joint-preserving surgery

Six studies reported joint-preserving surgical interventions in 44 patients aged 8–54 years [20, 23, 30, 36, 38, 41]. Two included both adults and children [20, 41], while the remainder focused on paediatric cohorts [23, 30, 36, 38]. Procedures comprised osteotomies, core decompression, cementoplasty, and vascularised bone grafting.

Ebong and Kolawole treated five children with Perthes-like AVN using rotational osteotomies and hip spica immobilisation, though operative details and rehabilitation protocol were limited [20]. Sene et al. also performed osteotomies in three patients who later required arthroplasty [38]. Akakpo-Numado et al. reported four children with Ficat stage IV disease managed with subtrochanteric varus osteotomy, followed by cast immobilisation and prolonged Thomas splinting for an average of 12 months [30]. Onyemaechi described a single stage-III case treated with an intertrochanteric osteotomy, but with limited details [23].

Bahebeck et al. performed core decompression in eight hips (six stage I, two stage II) [41]. Sene et al. treated six patients with either decompression alone or combined with cementoplasty [38]. Wu et al. reported the most technically advanced approach, offering vascularised iliac bone grafting to twelve patients aged 11–22 years [36]. Using an ilio-inguinal approach, necrotic bone was excised and replaced with a vascularised graft; patients underwent traction for 1-3 months before progressing to partial weight-bearing.

#### Definitive surgeries for end-stage disease

Fourteen studies, involving 207 patients, reported arthroplasty as the primary intervention (Tables 1 and 2) [17–19, 21, 23–25, 28, 29, 38, 41, 43–45]. Most procedures were total hip replacements (THR), although isolated cases of bipolar hemiarthroplasty, excision arthroplasty, and tibiotalar arthrodesis were also described [23, 41, 45]. Late-stage presentation predominated, hence salvage surgery was very prevalent. The vast majority were adults, with only four reports of THR in adolescents [38, 41, 43, 44]. Overall, 86% of patients had the SS genotype, likely reflecting more severe skeletal involvement.

Uncemented fixation was most frequently employed, with only two studies describing cemented implants [28, 38]. Alonge and Shokunbi favoured cementless components to conserve acetabular bone stock for potential revision surgery [29], while Lawal et al. proposed uncemented bipolar hemiarthroplasty as a cost-effective alternative to THR, requiring less expertise [45].

Surgical approaches varied based on individual patient characteristics, surgeon preference, prior surgical history, and anatomic considerations. Direct lateral was most common [17, 19, 21, 29, 43], with direct anterior [24], posterior [38], anterolateral [18], and combined techniques [25] also described. Lombardo et al. preferred the anterior approach for providing better short-term stability of the prosthetic components and to facilitate bilateral cases in the supine position. However, one intraoperative fracture required conversion to a direct lateral approach [24]. Manfroni et al. employed a lateral approach for one-stage bilateral THR to maintain extensibility in case of intraoperative complications, as intraoperative imaging was unavailable [25].

Adjunctive procedures included adductor tenotomy in patients with severe deformity, limb length discrepancy, pelvic obliquity, or hip contractures [29, 43]. Technical challenges were consistently encountered. Canal sclerosis and obliteration, along with poor bone quality and severe anatomical distortions, impeded femoral reaming, requiring smaller components and careful saline irrigation during reaming to prevent thermal injury [18, 21]. Ekere et al. and Katchy et al. documented iatrogenic proximal femoral fractures requiring cerclage wiring [18, 19]. To minimise this risk, Ugbeye et al. recommended in situ femoral neck osteotomy to reduce torsional stress during difficult dislocations [17]. Acetabular floor defects further compromised component seating and fixation, necessitating bone grafting in some cases [18, 38].

Collectively, these reports illustrate the significant technical challenges of arthroplasty in SCD-related AVN within resource-limited African orthopaedic centres. Across multiple studies, careful preoperative templating, optimisation, and meticulous soft-tissue handling were repeatedly emphasised as critical for minimising complications.

### Treatment outcomes and barriers

#### Conservative management

Conservative management produced favourable outcomes primarily in younger patients with early-stage disease [30, 37, 39]. Ndugwa reported clinical and radiological improvement in 69% of adolescents (mean age 17) with stage II disease treated with 6–12 weeks of traction and spica immobilisation. However, prolonged inpatient care was required [39]. Akakpo-Numado et al. similarly observed favourable results in six of eight patients (mean age 14) with stage III disease, who were followed up for nine years. They defined a good outcome as pain resolution or a stable, painless limp with radiological reconstitution [30]. Ebong observed symptomatic improvement in 22 patients (mean age 16) treated with alternating hip spica and knee cylinder, although radiological healing was inconsistent [37].

In contrast, outcomes in adults were poor. In a 75-patient cohort with a broader age range of 8–54 years (mean age 20.8), only five (6.7%) showed improvement, while most deteriorated [20]. Similarly, only three of 13 patients (23%) aged 16–51 years improved with conservative therapy [41]. Reported complications included joint stiffness, disuse atrophy, and pressure sores from prolonged immobilisation, while one unrelated mortality was attributed to chest infection [37, 38, 41]. Overall, these findings reinforce the limited efficacy of non-operative strategies in older patients and advanced stages.

#### Surgical interventions

Outcomes of joint-preserving surgery were variable but generally favourable in small cohorts. Ebong and Kolawole reported partial femoral head restoration in five patients, followed up for 6 to 24 months after rotational osteotomy, with one patient achieving normal radiographs [20]. Akakpo-Numado et al. also observed good results in four children following varus osteotomy [30]. Core decompression provided clinical but not radiographic benefit in seven of eight patients in Bahebeck’s series [41]. Wu et al. reported the most robust results: twelve adolescents undergoing vascularised iliac grafting for stage III–IV disease demonstrated Harris Hip Score improvements from 75 to 90 at two years, without revision [36]. Across studies, definitive surgical management for end-stage AVN generally resulted in favourable outcomes, with most patients reporting improved pain, mobility, and function at 6–30 months of follow-up. Early mobilisation was common; several authors documented ambulation within one day and discharge by day five [21, 24, 25]. Lawal et al. demonstrated significant pain reduction (from a Numeric Rating Scale score of 6 to 3) and an increase in walking distance (from 172 m to 614 m) [45].

Functional scores showed consistent gains. Ugbeye et al. reported good or excellent Harris Hip Scores (HHS) in 97% of patients [17]. Katchy et al. and Alabi et al. similarly documented substantial improvements in HHS and Oxford Hip Scores [18, 43]. Sene et al. highlighted genotype-specific differences, with excellent THR outcomes in 84% of SC versus 46% of SS patients, who also had higher complication rates (16% vs 2.6%) [38]. Beyond the hip, Bahebeck et al. reported complete pain relief and radiographic fusion after ankle arthrodesis [41].

Complications were frequent but varied. Superficial surgical site infections occurred in 4.8% to 20% of patients across the series, all of which were managed conservatively [18, 29, 43]. More serious complications included prosthetic joint infection, requiring revision in one case, and deep infection in two cases, which were successfully treated [38, 45]. Implant malposition and loosening necessitating early revision were also described, as were limb length discrepancies of 2–5 cm [17, 19, 38, 43]. Overall, arthroplasty offers substantial functional benefit in advanced AVN but is technically demanding, with outcomes influenced by genotype, surgical expertise, and anatomical severity.

#### Barriers

Systemic, patient-level, and resource-related barriers hamper management of AVN in SCD across African settings. The most prominent is late presentation: 85% of patients presented at Ficat stage III–IV, precluding joint-preserving options. Delays exceeding two decades, as reported by Ekere et al. and Ebong, reflect inadequate surveillance, limited referral systems, and poor awareness among the general population [19, 37].

Diagnostic limitations further compound delays. Advanced modalities such as MRI or CT were used in fewer than one-fifth of studies, with most centres relying solely on radiographs. Ben Dridi et al. highlighted 27% cases missed on X-ray, with some patients misdiagnosed as inflammatory arthritis, leading to inappropriate corticosteroid use, accelerating progression [16]. This reliance on insensitive tools hinders early intervention. N’Dri et al. demonstrated that CT identified lesions missed on plain films, and recommended CT scanning when magnetic resonance imaging or scintigraphy is unavailable in resource-constrained settings [33].

Resource constraints also limit surgical care. In Cameroon, Bahebeck et al. reported that patients were referred abroad for arthroplasty due to a lack of local expertise [41]. Financial barriers are widespread; Ndugwa noted that inpatient costs exceeded available budgets, while families frequently declined surgery due to prohibitive expenses [39]. High out-of-pocket payments in health systems with limited insurance coverage exacerbate inequities.

Cultural and social factors further delay timely treatment. Akakpo-Numado et al. and Onyemaechi et al. reported widespread reliance on traditional healers and bone setters, leaving patients financially depleted and presenting with advanced disease [23, 30]. Such patterns undermine trust in formal health systems and divert patients away from effective early care.

Together, these barriers create a cycle of late-stage disease, limited access to advanced diagnostics and surgical expertise, and unaffordable care. Tackling them requires investment in imaging infrastructure, affordable surgical services, community education, and organised referral pathways to improve musculoskeletal outcomes in this high-risk, vulnerable population.

### Discussion

This scoping review provides an overview of current practices in managing SCD-related AVN across Africa, synthesising evidence on surgical and non-surgical approaches while highlighting the influence of resource constraints, health system capacity, and socioeconomic factors. The findings emphasise not only the predominant treatment patterns but also persistent gaps in early diagnosis and access to joint-preserving interventions, underscoring the need for targeted improvements in care and research.

The evidence base remains limited. Most studies were small, descriptive, and heterogeneous in design, with inconsistent reporting of surgical techniques, rehabilitation protocols, follow-up, and outcomes. The largest series, published in 1986, involved 75 patients. Despite a modest rise in publications since the early 2000s (Figure 2), Africa contributed less than 10% of the global literature on SCD-related AVN, as determined by a PubMed bibliographic search, despite carrying over 80% of the disease burden. This research gap hampers the availability of locally relevant evidence to inform policy and practice. An additional limitation of this review is that seven of the included studies were conducted in francophone countries and required translation, which raises the possibility of subtle misinterpretations.

Late presentation was the dominant pattern, with 85% of cases diagnosed at Ficat stage III or IV, and delays sometimes exceeding two decades. Reliance on plain radiographs and limited access to MRI – rarely available and affordable despite being the gold standard – restricted early detection and joint-preserving options. Onyemaechi et al. emphasised the importance of affordable MRI in improving timely diagnosis and expanding the use of conservative or reconstructive strategies [23]. Disease expression also differed from earlier reports that suggested higher AVN prevalence in SC genotypes [46–49]. This review found that AVN was more common among SS patients, likely reflecting improved survival and higher skeletal complication rates, supporting Chung’s suggestion that earlier reports of higher SC incidence reflected their better life expectancy [50]. Gender differences, sometimes attributed to physiological stressors such as pregnancy or biomechanical strain, were inconsistently reported [20, 22, 39].

Conservative management demonstrated benefit in children with early disease but was largely ineffective in adults. Joint-preserving surgery yielded variable results, with vascularised iliac grafting showing the most encouraging outcomes; however, resource demands may limit scalability. Arthroplasty emerged as the dominant definitive treatment, producing consistent functional improvements but posing technical challenges related to canal sclerosis, bone fragility, and deformity. Outcomes differed by genotype: SC patients achieved better recovery and fewer complications than those with SS disease.

Comparison with high-resource settings highlights striking inequities. Elsewhere, MRI surveillance enables early detection and integration of joint-preserving interventions, including cell-based therapies and structured physical therapy [9, 51–53]. None of these were reported in African studies, reflecting infrastructural constraints and global disparities in translating advances to high-burden, resource-limited settings. Patients in Africa, where the burden is highest, have the least access to evolving interventions.

### Conclusion and recommendation

This scoping review highlights the considerable burden of late presentation with advanced-stage disease, which limits the role of joint-preserving interventions and leaves salvage procedures, such as arthroplasty, as the predominant management options. Although surgical outcomes are often favourable, these procedures are technically challenging, resource-intensive, and associated with a high risk of complications in this population.

The available evidence suggests that conservative management can be effective in early-stage disease and younger patients, but its success is limited in advanced cases. Importantly, there is no evidence that cell-based therapies and physical therapy – now established in other contexts – have been integrated into practice within Africa. There is considerable variability in reporting standards, with notable under-reporting of key variables.

Addressing these gaps requires a multipronged strategy. First, earlier detection and organised referral pathways must be prioritised and facilitated through structured musculoskeletal surveillance in SCD clinics, effectively collaborating with SCD advocacy groups, and by providing broader, affordable access to MRI, or, where unavailable, CT. Second, surgical capacity must be expanded for both joint-preserving and arthroplasty services through training, infrastructure investment, and affordable access to devices. Third, multicentre collaborative studies are needed to generate high-quality, locally relevant evidence. These studies should adopt standardised outcome measures, stratify by age, stage, and genotype, and incorporate cost-effectiveness analyses relevant to resource-constrained contexts. Ultimately, translational research examining feasible adaptations of biologics, regenerative techniques, and physiotherapy is crucial to bridging the gap with global practice, while developing a mechanism to mitigate the high out-of-pocket healthcare expenditures for this vulnerable group of patients.

## Data Availability

All data generated or analysed during this study are included in this published article.
